# A feasibility study investigating cortical hemodynamic changes during infinity walk with fNIRS

**DOI:** 10.1016/j.ibneur.2024.01.003

**Published:** 2024-01-29

**Authors:** Haroon Khan, Noman Naseer, Peyman Mirtaheri

**Affiliations:** a Department of Mechanical, Electronics, and Chemical Engineering, Oslo Metropolitan University, Pilestredet 46, 0167 Oslo, Norway; b Department of Mechatronics and Biomedical Engineering, Islamabad, Pakistan

**Keywords:** Functional near-infrared spectroscopy (fNIRS), Figure-of-8 Walk, The Infinity Walk, Pronation, Footwear, Walking Pattern, Cortical Hemodynamics

## Abstract

This study seeks to explore the correlation between cortical activation and the Infinity Walk pattern, examining how the influence of foot overpronation and footwear may impact motor control. Functional near-infrared spectroscopy (fNIRS), a portable and user-friendly neuroimaging technique, was used to measure hemodynamical changes in six individuals with non-critical pronation degrees. Participants perform the Infinity Walk under various footwear conditions while wearing an fNIRS portable imaging device. Results indicate a consistent hemodynamic pattern in both hemispheres during the Infinity Walk, with no significant differences observed across subjects and footwear conditions in the prefrontal cortex (PFC), pre-motor area, the supplementary motor cortex (PMA & SMC), the primary motor cortex (PMC), and Wernicke’s area (WA). The impact of pronation and footwear on motor control remains inconclusive due to inconsistent hemodynamic patterns. Notably, the activation patterns in Broca’s area (BA) and the temporal gyrus (TG) differ significantly from other brain regions. The balanced hemodynamic responses in the bilateral hemispheres may be attributed to the Infinity Walk’s inherent walking pattern. These findings indicate a need for further investigation into the Infinity Walk to examine the similarities and distinctions in activation patterns within specific brain regions. Additionally, the impact of pronation necessitates more substantial experimental research to establish a correlation between pronation and cortical hemodynamics.

## Highlights

• Study links Infinity walk to cortical activations.
• Infinity walk atributes to well-balanced brain activations in both hemospheres.
• Hemodynamic activations obsersed in prefrontal, sensomotoric region were synchronized except part of temporal gyrus.

## 1 Introduction

Functional near-infrared spectroscopy (fNIRS) is a non-invasive neuroimaging technique that is influenced by neurovascular effects in local brain regions using light wavelengths within near-infrared spectra, i.e., 700 nm −1100 nm (Izzetoglu et al., 2005). Among non-invasive brain signal acquisition technologies, fNIRS is a non-invasive brain sensing technology with several advantages such as ease of use, portability, spatial resolution, and less prone to motion artifacts compared to other portable modalities (Khan et al., 2021a). The measurements obtained from fNIRS can be instrumental in enhancing therapeutic interventions, optimizing pain management strategies, and developing more effective rehabilitation and therapy plans (Wang et al., 2023, Mihara and Miyai, 2016. This study uses fNIRS to measure changes in brain activity during a semi-challenging walking pattern known as the Infinity Walk, which was initially designed to improve motor function in patients with neurological disorders such as stroke. The work helps give an insight into cortical activity during such therapeutic exercises to monitor the effectiveness of the intervention over time on the brain.

Gait speed and variability were associated with the strength of functional connectivity of different brain networks (Lo et al., 2017). Gait performance parameters to assess the risk of falls, injuries, or related cognitive function are evaluated in many ways, such as a change in walk speed, patterns, or dual-tasking conditions (Hamacher et al., 2015). The Infinity Walk, introduced by psychotherapist Deborah Sunbeck in the mid-1980s (Sunbeck, 1996, Szymanski, 1997, is a figure-of-8 walk designed to enhance motor, sensory, and cognitive abilities, promoting a balanced gait and improved neuromuscular coordination (Sunbeck, 2006). This therapeutic approach is selected for its natural incorporation of left-right foot placement shifts and ankle joint rotation, facilitating coordination between both sides of the body. The alternating movements of the body’s left and right sides elicit neuronal activation in both hemispheres. Materials on the Infinity Walk claim that it enables us to leverage our bodies as instant biofeedback systems. In collaboration with trained Infinity Walk observers, this approach provides valuable insights for enhancing motor control and postural balance (Sunbeck, 2006, 2002). Growing evidence has suggested that gait training has improved stroke patients’ balance outcomes (Lyu et al., 2023). The Infinity Walk (also called Figure-of-8 walk) represents ordinary walking skills, incorporating straight and curved paths. The sharp curved ends in the Infinity Walk can even pose challenges for healthy individuals, and in particular for those with motor challenges. However, it has the potential to provide valuable insights into the progression of diseases and improve balance, safety, autonomy, and overall quality of life compared to walking in a straight line (Belluscio et al., 2020). The Infinity Walk has been reported as a valid measure of walking skill, encompassing various aspects such as physical function, movement control (step width and length variability), gait speed, and confidence, particularly in older populations (Hess et al., 2010). The Infinity Walk also offers unique attributes for assessing gait, including heel-to-foot time and reaction time (Odonkor et al., 2013). Furthermore, it has been established as a valid indicator of motor skill in people with Parkinson’s disease (Lowry et al., 2022) and also an assessing indicator for the mobility of lower limb amputation (Schack et al., 2021). Increasing the complexity of the Infinity walk by incorporating dual-tasking can also aid in predicting falls among older adults (Nualyong and Siriphorn, 2022). The support rail design of the Infinity Walk would help in enhancing the postural stability, gait, and navigational performance of not only the physically weak participant but also the visually impaired population (Rogge et al., 2021). The literature on cortical activation during the infinity walk is limited, and to the best of our knowledge, except our previous report on the effective connectivity of brain regions during the infinity walk (Khan et al., 2023a). The brain activation investigated in previous studies and our current study includes the prefrontal cortex (PFC), pre-motor area, the supplementary motor cortex (PMA & SMC), the primary motor cortex (PMC), and Wernicke’s area (WA) (Hamacher et al., 2015). Our previous findings reveal robust bi-directional intra- and inter-hemispheric functional connectivity between the brain regions of interest (ROI), highlighting the potential for motoric learning, neuroplasticity, and neuro-rehabilitation (Khan et al., 2023a).

The main goal of this study is to examine cortical activation patterns during the Infinity walk, which involves both straight and curved paths. Additionally, we aim to explore the impact of pronated walking patterns and the use of anti-pronating shoes in correcting posture on cortical activation. Furthermore, we seek to investigate how pronation affects motor control in this study. We will also delve into the effects of different types of footwear, including anti-pronating shoes, and further explore the lateralization of motor control and brain hemodynamics. The recorded brain region is classified into six ROI for an in-depth understanding. The forthcoming sections cover the methodology (section 2) adopted, results and discussion (section 3), and conclusion (section 5) of the work.

## 2 Materials and methods

### 2.1 Participants’ selection and ethical consideration

The participants (one male and five females) were in a good physical and mental state to perform the walking without any extra health concerns or complications. We set the age range of the subjects to 20–40 years old, as there can be age-bound statistical differences between young adults and higher age groups in pressure distributions and skin sensitivity Machado et al. (2016). Further, it includes non-critical degrees of pronators that the untrained eye cannot easily detect. The method for determining pronation angle is described in section 2.2. The experimental protocol was approved by REK (Regional Committee for Medical and Health Research Ethics, reference no. 322236) and NSD (Norwegian Center for Research Data, reference no. 751430). The experiment was conducted according to the latest Helsinki Declaration.

### 2.2 Pronation angle calculation and selection of different footwear

**Fig. 1:**
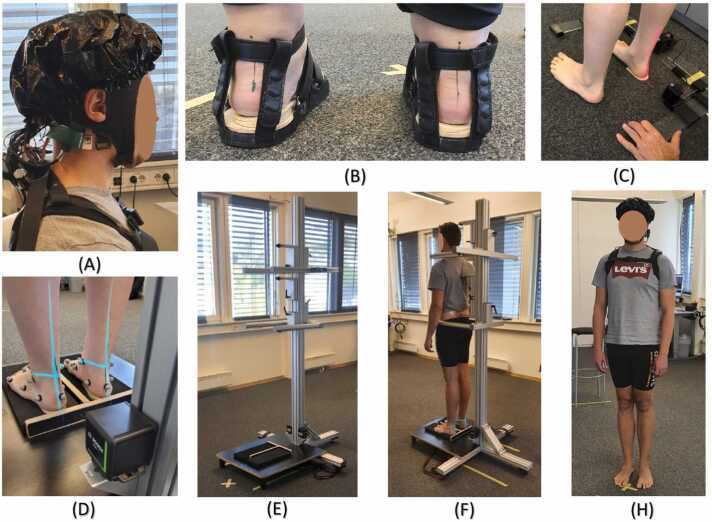
Experimental Setup: (A) Demonstration of fNIRS equipment on participant head (B) Wedged flat sandals: Medial heel wedge angle to correct the pronation in flat sandals (C-F) Achilles laser making setup with posture corrector (H) Participant wearing portable fNIRS device backpack.

Non-critically pronating subjects were selected for the experiment. These subjects were young and adjusted their pronated pattern, so it was not easy for the common humans to determine the degree of pronation. Using an Achilles marker and laser, the Achilles tendon and the vertical line to the ground can determine the degree of pronation. A custom-made Achilles marker by Gaitline AS is used to mark the center line of the Achilles tendon while the subject lies face down. Then, the vertical lasers in the Achilles laser tool are matched to this line as shown in Fig. 1D. In the frontal plane, the instrument is a frame that is placed around the subject’s foot, indicating the leg’s vertical axis. The laser line is aligned with the traced mark by placing medial wedges of the appropriate degree under the heel bone. Thus, the degree of correction needed is determined for both feet. The use of the Achilles laser is illustrated in Fig. 1. The degree of pronation calculated is shown in the supplementary material (see table 2 in supplementary materials). In Fig. 1A shows the fNIRS setup, Fig. 1B shows the flat sandal with wedges inside, Fig. 1C-F demonstrate the Achilles tendon marker and the postural corrector setup.

Participants completed the Infinity walk under five different footwear conditions (see Table 1). The first condition involved walking barefoot, serving as the baseline since it represents the most common and natural walking condition. The second and third conditions involved walking in sandals. The rationale for using sandals was twofold: first, to minimize the impact of upper shoe coverings on somatosensory activation, and second, to observe proprioceptive feedback for balance maintenance. Additionally, sandals were employed to facilitate the precise tracking of markers (utilized by the motion capture system) placed on the feet during walking. It’s important to note that the results from the motion capture system are not included in this study. The fourth and fifth conditions involved walking in one’s own shoes and walking in anti-pronating shoes with (SGL technology shoe, GAITLINE AS). These conditions were implemented to investigate differences in postural control between the two footwear options. Furthermore, the angle of the calcaneus and the ground when it touches the ground has to be as close to a normal angle as possible. It can dangle in the air, which does not influence the gait and balance. Any deviation of the angle between the calcaneus bone and the ground would influence the forces that the skin between the bone and the ground experiences. If part of the sensory information is coming from the heel skin (Horak, 2006), then these deviations will influence the sensorimotor control. The infinity walk would, in particular, challenge these deviations in the curvy parts. Thus, the infinity walk would be an ideal experimental paradigm to challenge the biomechanical motions while measuring brain activations in a repetitive pattern.

**Table 1:** List of conditions tested during Infinity walk. Tests were performed in the same order as 1 to 5.

Conditions	Descriptions
1	Walking barefoot on the carpeted floor of the laboratory.
2	Walking with flat inner and outer sole sandals are specifically designed for this experiment.
3	Walking with corrected pronation angle by placing required medial heel wedge inside flat sandals
4	Walking with personal shoes, which is used daily by the participants
5	Walking with SGL technology shoes (anti-pronating) designed by the GAITLINE AS, Norway

### 2.3 Experimental design

**Fig. 2:**
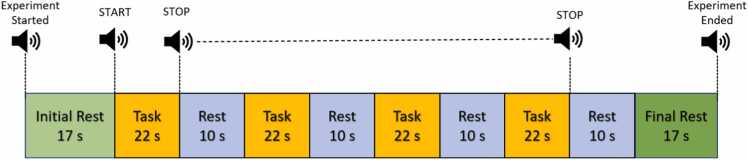
Experimental paradigm: Task represents the Infinity walk with different footwear conditions Table 1. Each condition was analyzed using the same paradigm.

Before the initiation of the experiment, participants were given explicit instructions about the experimental protocol, and demos were conducted to familiarize them. Before fNIRS experimentation, Achilles was marked, and the degree of pronation was determined as described in the section 2.2. The experimental protocol consists of an initial and final rest for 17 s (in a standing position), a task period of 22 s (one complete infinity walk in a single-task block), a rest time of 10 s between consecutive walks, and starting with the central point of pattern eight as shown in Fig. 2 and (see fig. 7 in supplementary materials). Since the gait speed and variability affect the brain’s functional connectivity, we asked the participants to walk with the preferred walking speed to optimize oxygen uptake and complete one round of pattern eight in the task period using walking economy (Lo et al., 2017, Gjovaag et al., 2018). Since walking difficulty in walking increase the energy expenditure (oxygen uptake *VO*_2__*max*_) (Starholm et al., 2016). The preferred walking speed is also behavioral and distance affected (short or long), and energy-optimal walking speed for the shorter distance is lower (Seethapathi and Srinivasan, 2015). One complete Infinity walk was performed in each task period (on average 20 s) and repeated four times in a single experimental run. Auditory stimuli of ‘START’ and ‘STOP’ were announced to guide the participant. The experimental paradigm is shown in Fig. 2.

The fNIRS optode placement tool, known as fNIRS Optodes’ Location Decider (fOLD v2.2), was employed to position the optodes over the ROI based on the Brodmann areas (see table 3 in supplementary materials) (Morais et al., 2018). Optodes were placed over the motor and somatosensory cortex according to the 10–10 international system using the ICBM 152 head model (Fonov et al., 2011) (see fig. 8 in supplementary materials). Six different regions (ROI) of interest were observed, i.e., prefrontal cortex (PFC or PF), Broca’s area (BA), pre-motor area & supplementary motor cortex (PMA & SMC), the primary motor cortex (PMC), Wernicke’s area (WA)and temporal gyrus (TG) (see table 3 in supplementary materials). A continuous-wave fNIRS device, NIRSport 2 (NIRx Medizintechnik GmbH, Germany), was used to acquire the data with a sampling frequency of 10.2 Hz. The device uses two infrared light wavelengths (760 nm and 850 nm) with 16 emitters and 14 detectors. Short channel data were recorded during the experiment, but due to the poor quality of the data, it was not used in the analysis.

### 2.4 Signal processing

**Fig. 3:**
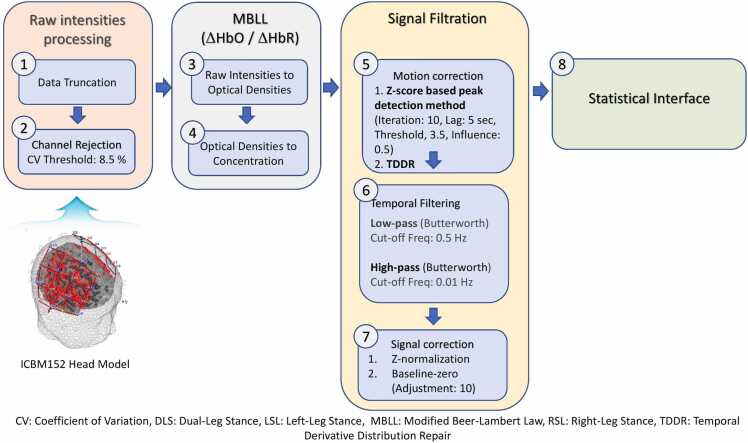
Data processing flow: The sequential flow of data processing till statistical analysis.

The data processing was performed with Satori v1.6.4 (NIRx Medizintechnik GmbH, Germany), while Matlab® was employed for visualization. The initial step in analyzing the data was to remove before and after the last stimuli data, as they could significantly impact the statistical analysis due to various participant movements or activities, etc. As the participant walked during the experiment, many motion artifacts were observed in the data. Therefore, motion artifact removal steps were carefully performed to clear all possible contamination. A summary of the signal processing pipeline is shown in Fig. 3.

Different approaches, such as the scalp coupling index (Pollonini et al., 2014) and coefficient of variation (CV) (Morais et al., 2017), were considered. Both approaches are robust to channel rejections and reject approximately the same channels. However, CV was more efficient in our case and fit the single processing pipeline chosen for this experiment. CV is defined as CV(%) = 100 × standard deviation (raw data) / mean (raw data) (Zimeo Morais et al., 2018). The CV value was set to 10 % after testing various CV values to compromise between data quality and the maximum number of channels in the data. Two channels (CH34, CH35) were rejected for Participant 1 performing a barefooted walk. The change in the raw intensities was converted into optical densities and then into concentration changes (oxygenated hemoglobin (HbO), de-oxygenated hemoglobin (HbR) using modified Beer-Lambert law (MBLL).

The data contain motion spikes due to motion and head movement while performing the Infinity walk. We carefully processed the data to remove these peaks of motion artifacts. A sudden peaks (spikes) detection algorithm using z-scores (van Brakel, 2014) was used to remove sudden spikes in the data. The algorithm takes four inputs, i.e., iterations, lag, threshold, and influence. Iteration is the number of repetitions of the algorithm, and lag (sec) is the lag moving window used to smooth the data (last ’n’ sec to data before the peak). At the same time, the threshold is the z-score at which the algorithm signals, and the influence (value range between 0 and 1) determines the influence of signals on the detection threshold of the algorithm. The algorithm parameters were set to iterations (= 10), lag (= 5 sec), threshold (= 3.5), and influence (= 0.5). The correction of detected spikes was performed using monotonic interpolation (Steffen, 1990). Furthermore, to remove the remaining spikes and baseline shifts, a robust regression-based temporal derivative distribution repair motion correction algorithm (Fishburn et al., 2019) was applied, allowing for automatic correct motion artifacts in fNIRS data. The method is based on robust regression and allows for removing baseline shifts and spike artifacts. The data processing pipeline is shown in Fig. 3.

### 2.5 Averaging analysis

**Fig. 4:**
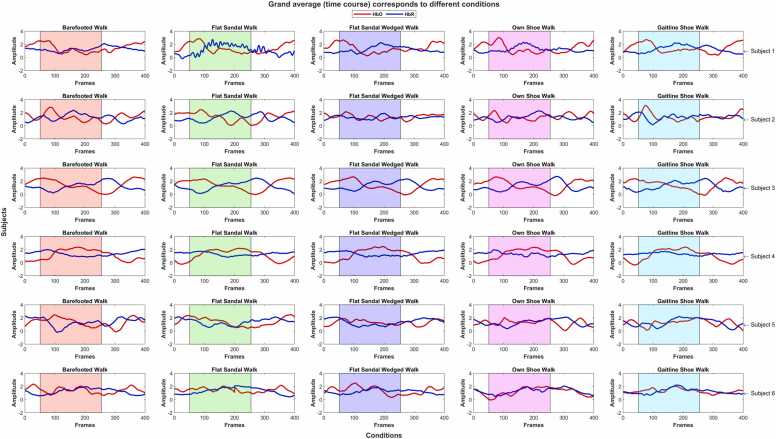
Grand channel averaging over both hemispheres: columns represent different conditions while rows represent the subjects. The shaded region shows the stimuli duration. Changes in amplitude are in μM.

Channel averaging involves several steps, including the calculation of the average response across the trials for each condition, subject, and brain ROI. The resulting average responses are presented as overall cortical hemodynamic responses, encompassing both hemispheres across different subjects and tested conditions as shown in Fig. 4). Additionally, individual hemisphere responses were computed (see figs. 9 and 10 in supplementary materials). These individual hemisphere responses were derived through a grand spatial averaging process, averaging data from channels specific to the participant’s left, right, and both hemispheres for each condition. The events were averaged from 5 s before stimulus onset to 32 s after, encompassing the 22-second task duration and an additional 10 s of post-rest duration. This extended duration was chosen to observe the settling of the hemodynamic response.

**Fig. 5:**
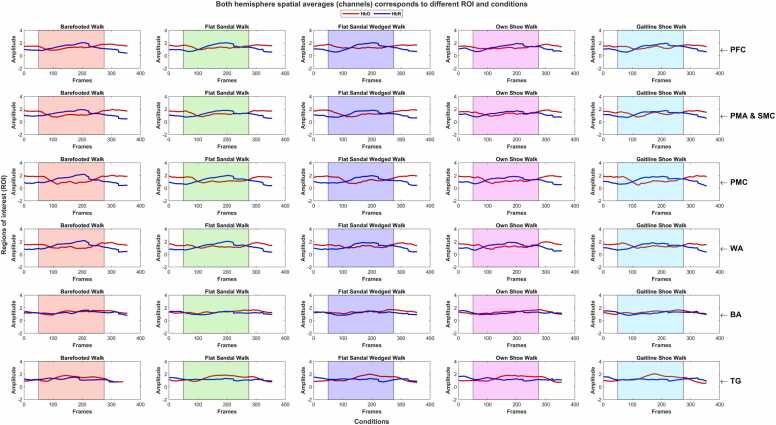
Grand averaging ROI both hemispheres: columns represent different conditions while rows represent the ROI. The shaded regions The shaded region shows the stimuli duration. Changes in amplitude are in *μM*. PFC: Prefrontal cortex, PMA & SMC: Pre-motor and supplementary motor cortex, PMC: Primary motor cortex, WA: Wernicke’s area, BA: Broca’s area, TG: Temporal gyrus. Further details about the ROI can be found in (see table 3 in supplementary materials).

The other type of grand average response involves averaging over the ROI for each condition. After the event, we extracted the average response per participant for each channel and each condition. Subsequently, we averaged together the channels corresponding to the respective ROIs as indicated in table 3 to generate an ROI average waveform for each participant. It’s important to note that the number of channels may vary due to channel rejection within this average for each participant and ROI. These ROI average waveforms were then utilized to create a time series visualization, which displays the average signal across participants for each condition and ROI as shown in Fig. 5. This visualization serves to summarize the results of the group-level averaging analysis. For this particular type of event average, calculations were performed from 5 sonds before stimulus onset to 30 s after.

**Fig. 6:**
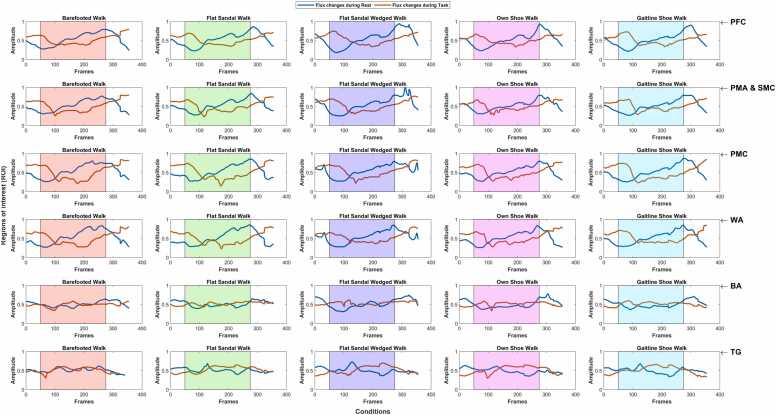
Blood flow changes over ROI in both hemispheres: columns represent different conditions while rows represent the ROI. The shaded regions The shaded region shows the stimuli duration. Changes in amplitude are in *μM*. PFC: Prefrontal cortex, PMA & SMC: Pre-motor and supplementary motor cortex, PMC: Primary motor cortex, WA: Wernicke’s area, BA: Broca’s area, TG: Temporal gyrus. Further details about the ROI can be found in (see table 3 in supplementary materials).

For testing the hypothesis in the group averaged result (among different participants, conditions, and ROI), a one-way ANOVA test was performed to see the variances among the different conditions and ROI. The hypothesis tested was if there is any significant difference between different walking conditions corresponding to subjects and brain regions. If significant variance among the conditions was observed, pair-wise t-tests were performed to determine the degree of difference between the groups. Blood flow changes which are defined as *Bloodflow* = Δ*HbO*∕Δ*HbT* were also calculated across the ROI and conditions (Yamaguchi et al., 1999). The averaged blood flow changes over the change over the ROI in both hemispheres are shown in Fig. 6 (see figs. 13 and 14 in supplementary materials).

## 3 Results and discussion

The results are discussed sequentially from three different perspectives to observe the effect of Infinity Walk against other conditions Table 1 and ROI. First, the impact of subject-wise changes in Δ*HbO* and Δ*HbR* were plotted against different conditions tested. Second, the effect of varying footwear/conditions on brain hemodynamics and changes in addition to ROI were observed. Lastly, the blood flow in the different ROI against various footwear conditions was plotted. Note all these conditions Table 1 were tested while the participant performed the Infinity Walk.

In Fig. 4 show the spatial average (channel average across both hemispheres) for all the subjects and condition Table 1. There is no statistically significant difference (*P* ≥ 0.05 *&* *F*_*crit*_≥*F*) in the Δ*HbO* and Δ*HbR* for each subject and all different walking conditions expect to walk with the shoe for subjects 5 and 6. The subject-wise variability of hemodynamic response is clearly demonstrated. However, the effect of other footwear on the hemodynamic response is insignificant. There are a few exceptions to these results; for subjects 5 and 6, the variation in Δ*HbO* was found statistically significantly different while performing the Infinity walk with shoes, i.e., own and GAITINE AS shoe (SGL technology shoes). For these two subjects, the Δ*HbR* changes across all the conditions were the statistically significant differences (*P* ≤ 0.05 *&* *F*_*crit*_≤*F*). The considerable difference in Δ*HbR* changes is also found in a few other conditions. The results revealed a mixed pattern of hemodynamics in response to different footwear conditions. These findings suggest that the footwear condition needs to be tested separately from the Infinity Walk to avoid the mixing effect of the Infinity Walk and footwear. Interestingly, both hemispheres are equally affected as a result of the walk-in Infinity pattern. By comparing the individual responses of Δ*HbO* and Δ*HbR* for each subject and respective conditions, we found no statistically significant difference in both hemispheres (see figs. 9 and 10 in supplementary materials). The results of the Infinity Walk study suggest the potential of eliciting similar cortical hemodynamic patterns in the hemispheres.

The channel averaging of Δ*HbO* and Δ*HbR* corresponding to ROI (see section 6 in supplementary materials) and conditions tested across the subjects are shown in Fig. 5. This type of analysis was to observe the behavior of different brain regions in the motor cortex and associated areas in response to the Infinity walk and different footwear conditions. By comparing the hemodynamic response (changes in Δ*HbO* and Δ*HbR*) in the PFC, PMA & SMC, PMC, and WA against different walking patterns, there is no statistically significant difference in activation Δ*HbO* except for the walk-in flat sandal (C3) which show differences in activation across these areas. The response of BA and TG areas are statistically significantly different activation patterns from the rest of the brain regions. A similar trend of activation was observed in both left and right hemispheres (see figs. 11 and 12 in supplementary materials). Also, looking into the Δ*HbO* and Δ*HbR* changes in individual condition was also similar in the brain regions such as PFC, PMA & SMC, PMC, and WA except for the BA and TG areas. While comparing the activation in the left and right hemisphere across all respective different conditions and ROI (PFC, PMA & SMC, PMC, and WA), the changes in Δ*HbO* and Δ*HbR* are not significant with few inconsistencies. In summary, the results do not demonstrate the effect of footwear on brain hemodynamic response in different ROI of interests. Interestingly, the PFC, PMA & SMC, PMC, and WA have similar cortical hemodynamic responses.

In Fig. 6 shows the blood flow changes over the ROI in spatial averaging over both hemispheres corresponding to each condition. The blood flow during the resting and task periods for each condition tested in the experiment was not statistically significantly different across the brain regions such as PFC, PMA & SMC, PMC, and WA. However, the blood flow in the BA and TG areas was different. The blood flow was consistent for the spatial average across the left and right hemisphere (see figs. 13 and 14 in supplementary materials) except the blood flow during the task period for conditions C3 & C4 in the right hemisphere. Similarly, the blood flow in the PFC, PMA & SMC, PMC, and WA was also not statistically different across each of the conditions with few exceptions. It is very clear the blood flow in PFC, PMA & SMC, PMC, and WA brain regions are similar, and there is similar activation between both hemispheres during the Infinity walk. The walking speed affects the metabolic demand, and energy-optimal walking speed is lower for short distances (Seethapathi and Srinivasan, 2015). The average speed during the Infinity walk was 2.01 ± 0.15*ft*∕*s* (0.61 *m*∕*s*) (see table 4 in supplementary materials), almost half of the average normal walking speed since the turn in the Infinity pattern controls the speed. But at the same time, turning during walking increases energy expenditure, which must also be considered during the Infinity walk (McNarry et al., 2017). In the future, both these parameters need to be considered.

The individual general linear model (GLM) is applied to investigate significant active channels and levels of activation corresponding to different conditions. Significant active channels (*p* ≤ 0.05) of Δ*HbO* with the corresponding t-value (see figs. 16 and 17 in supplementary materials). Since the resting time between the trail was not enough to settle the hemodynamics to baseline during the rest period, an overlapping increase of Δ*HbR* and decrease Δ*HbO* can be observed during the task period. Also, the level of significant activation cannot be correctly perceived from the result. However, the number of active channels corresponding to an ROI and condition can be identified, which helps understand motor cortex activation. The highest number of significantly active channels were found when the subject performed a barefooted walk, which is inconsistent with our previous study, which shows the lowest cortical activation in the motor cortex for barefooted walk (Khan et al., 2021b, 2023b). The reason for the difference might be the pattern of walking, i.e., walking in the Infinity pattern instead of straight walking. It could be that when walking barefoot, our brain receives more sensory information from the feet and better proprioception about our walking environment than when we wear excessively supportive and cushioned shoes (Saxby, 2011). In flat sandals, we found a similar number of activation channels. The number of active regions (channels) in other conditions was similar. The activation in ROI of interest varied from subject to subject, such as Subjects 1 and 3 having the highest active ROI compared to others. Overall, WA and PFC were found to be the highest and lowest active regions among all participants and tasks performed, respectively. Other regions show the same number of activations. The number of significantly active channels in the left and right hemispheres are similar except for PMA & SMC, which shows slightly higher activation in the left hemisphere compared to the right.

## 4 Limitations and future recommendations

It’s important to emphasize that this pilot study, aimed at observing cortical changes during the Infinity Walk, has specific limitations, particularly regarding its sample size. Consequently, making broad generalizations to a larger population may not be feasible. Regardless, the study bestows upon the pilot an understanding of the relationship between cortical activation and the Infinity Walk. Furthermore, it’s worth noting that the participants in this study were non-critically pronating individuals. To comprehensively assess the effectiveness of the Infinity Walk in various contexts, further research is required involving specific groups such as highly pronating subjects, older age groups, or individuals with lateral postural imbalances. Further experiments will, however, be carried out based on the results of this study. Despite a 10-second rest period between consecutive movements/tasks, the hemodynamics were not able to settle back to baseline completely, which overlapped the activation periods. Also, it makes it difficult to quantify the level of significant activation corresponding to a different condition. The rest time should be increased in future experimentation. Inclusion of a dual tasking (visual and arithmetic tasks) component would significantly enhance the experimental design, providing a more comprehensive understanding of the Infinity Walk’s efficacy in specific applications. For the in-depth lateralization study, pausing (long enough to settle the hemodynamics) at both ends of pattern eight makes it more understanding of bi-direction rotation effects on lateral control. When walking in the Infinity pattern, measuring the effects of different footwear and pronation is difficult. A straight line walk could better understand footwear and pronation effects. The need for future investigations to minimize the impact of other variables on cortical hemodynamics and concentrate solely on the effect and degree of pronation on cortical hemodynamics is apparent.

## 5 Conclusion

In conclusion, the findings of this study provide compelling evidence that the Infinity Walk exhibits unique characteristics in activating specific brain regions, demonstrating a positive impact on neurovascular activation with consistent activation observed in both hemispheres. A well-balanced activation pattern was observed in the right and left hemispheres, which is likely attributed to walking in the infinity pattern. However, it is challenging to draw definitive conclusions regarding the effects of footwear and pronation on brain hemodynamics and motor control. This is primarily due to the mixed effects of the Infinity Walk on cortical activation and the limited sample size, which did not yield consistent results for conclusive statements. Notably, the prefrontal cortex (PFC or PF), pre-motor area and supplementary motor cortex (PMA & SMC), the primary motor cortex (PMC), and Wernicke’s area (WA) exhibited similar responses across all subjects and conditions, except for Broca’s area (BA) and the temporal gyrus (TG). Improvements in the experimental paradigm are needed to further investigate the effects of footwear. Additionally, future research should aim to validate these findings in larger populations to ensure their robustness and generalization.

## CRediT authorship contribution statement

**Haroon Khan:** Conceptualization, Methodology, Software, Formal analysis, Investigation, Data Curation, Visualization, Writing - Original Draft, **Noman Naseer:** Methodology, Writing - Review & Editing, Supervision **Peyman Mirtaheri:** Methodology, Writing - Review & Editing, Supervision, Project administration.

## Conflict of Interest

The authors declare that they have no known competing financial interests or personal relationshipsthat could have appeared to influence the work reported in this paper.
