# Supplementary material for: A feasibility study investigating cortical hemodynamic changes during infinity walk with fNIRS
Source: IBRO Neurosci Rep. 2024 Jan 29;16:309–16. doi: 10.1016/j.ibneur.2024.01.003 (PMC10882108; doi:10.1016/j.ibneur.2024.01.003)
Supplement: Supplementary file 1 — Supplementary material [file mmc1.pdf]

A Feasibility Study Investigating Cortical Hemodynamic Changes during Infinity Walk with fNIRSThe Infinity Walk: A Promising Approach to Enhancing Motor Control and Balance - An fNIRS Study (Supplementary Material)

Haroon Khan[a], Noman Naseer[b], Peyman Mirtaheri[a], Haroon Khan, Noman Naseer, Peyman Mirtaheri

[c]*Department of Mechanical, Electronics, and Chemical Engineering, Oslo Metropolitan University, Pilestredet 46, 0167 Oslo, Norway*
[d]*Department of Mechatronics and Biomedical Engineering, Islamabad, Pakistan*

6. Experimental Information

The section contains supportive information related to the experiment. In fig. 7 show the demonstration of the Infinity walk along with the dimensions. The degree of the wedge on both sides of the show for each participant is shown in table 2. The optodes arrangement over the motor cortex is shown in fig. 8. Standard montage configuration in the Aurora fNIRS is used with short channels. The Brodmann region corresponding to each is shown in table 3 to precisely categorize regions of interest (ROI). The ROI are broadly classified into Prefrontal cortex (PFC), Pre-motor and supplementary motor cortex (PMA & SMC), Primary motor cortex (PMC), Wernicke's area (WA), Broca's area (BA), and Temporal gyrus (TG). Short channels data were collected but due to the bad quality of data from short channels, it was not considered in any stage of data analysis.

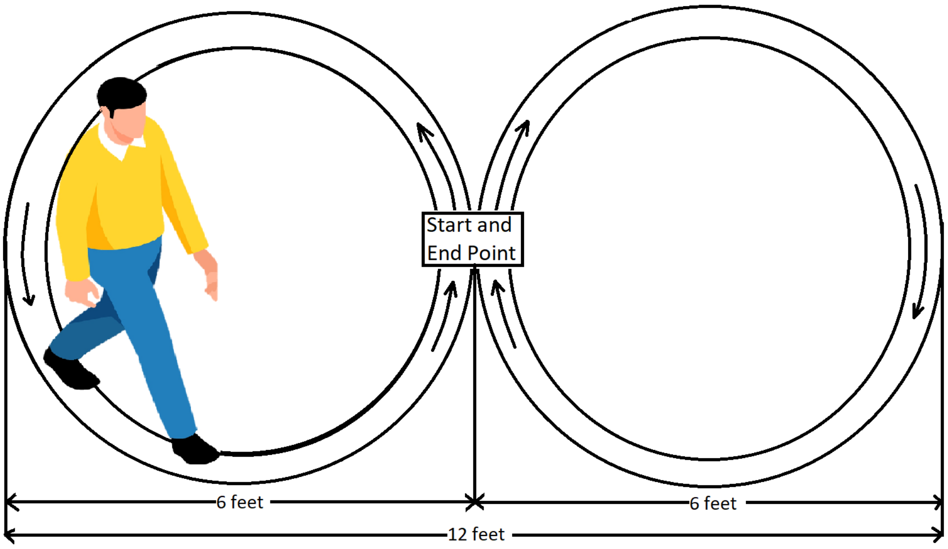

Figure 7: Demonstration of Infinity Walk (IW) pattern

7. Channel Averaging Results

The section includes channel averaging analysis performed to investigate the overall trend of activation in both hemispheres and different ROI.

*corresponding authors, peymanm@oslomet.no
Email addresses: haroonkh@oslomet.no (Haroon Khan[a]), noman.naseer@mail.au.edu.pk (Noman Naseer[b]), peymanm@oslomet.no (Peyman Mirtaheri[a])

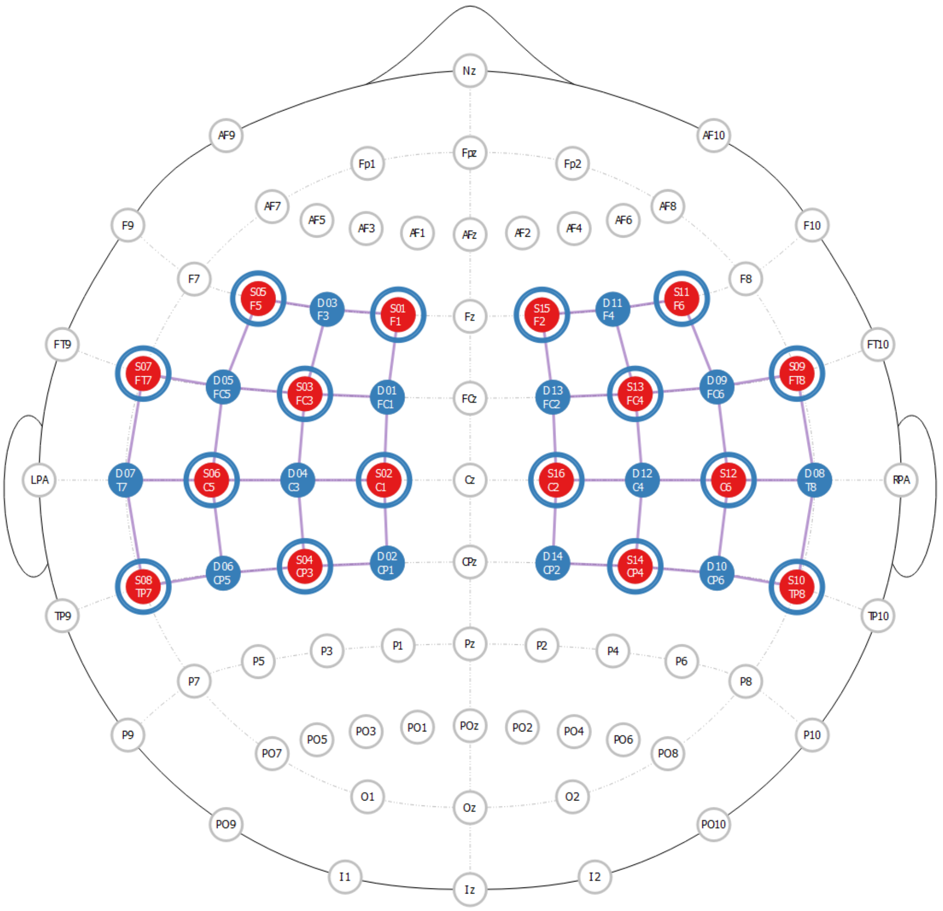

Figure 8: Optodes configuration over the region-of-interest

7.1. Event averaging

Channels of the left and right hemispheres are averaged together per subject and conditions tested regardless of ROI. The figs. 9 and 10 show the average ΔHbO and ΔHbR response over the left and right hemispheres, respectively.

7.2. Event averaging over regions of interest

Channels in the corresponding ROI are averaged on the left and right hemispheres for all subjects and conditions. The figs. 11 and 12 show the average ΔHbO and ΔHbR response over the left and right hemispheres, respectively.

7.3. Blood flow changes over the ROI

The blood flow change in the ROI at left and right hemispheres are shown in fig. 13 and fig. 14. respectively.

8. General Linear Model (GLM)

The General Linear Model is a supervisory approach that combines experimental design knowledge and signal morphology from a priori knowledge [38]. In the presence of physiological nuisance signals, this approach provides the best unbiased linear estimate of hemodynamic response to a series of stimuli. GLM serves as a standard statistical method in fNIRS to measure the changes in ΔHbO and ΔHbR. This study analyzes the time series ΔHbO data using GLM with the following model. The simplest form of the model is shown in Eq. 1 [39].

$$y = X\beta + e \tag{1}$$

Table 2: The medial heel wedges angles to correct pronation on both for each subject

Subjects	Left leg (degrees)	Right leg (degrees)
1	12	6
2	8	11
3	17	17
4	12	9
5	17<br>An additional 5 mm flat heel lift was added to the subject right-leg to balance the height of the legs.	17

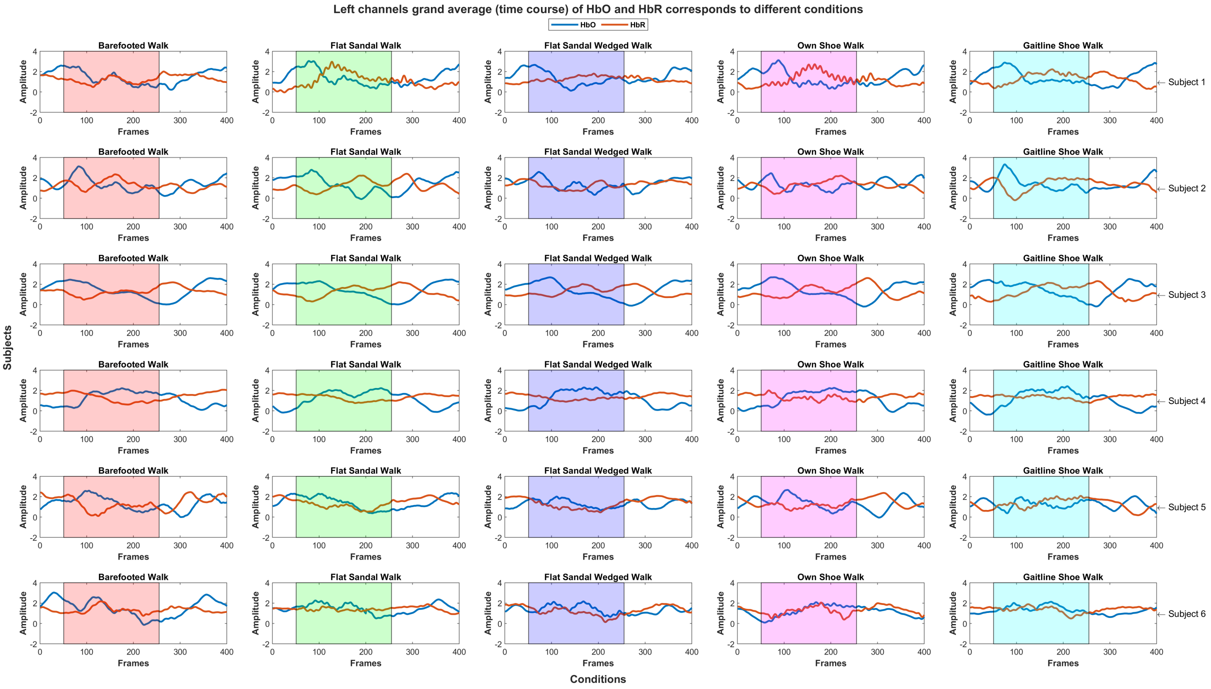

Figure 9: Grand averaging of left side channels

where $X \in R^{N \times M}$ represents the design matrix (M denotes the number of time points, and N represents the β dimension). The $\beta \in R^{M \times L}$ (where L is the number of channels) is the corresponding response signal strength for $\Delta HbO/\Delta HbR$ at the respective L channel. The error term is represented by e. The GLM fitting procedure finds the set of β values that explains the data with a perfect fit. The model's time course values are predicted by the linear combination of predictors provided by the data y and design matrix X as shown in Eq. 2.

$$\hat{y} = X\beta \tag{2}$$

The prediction of β value should be close to y, the measured values for a possibly good fit. With the equations rearranged according to the prediction, it becomes apparent that small error values (Eq. 3) lead to a good prediction.

$$e = y - X\beta = y - \hat{y} \tag{3}$$

It would seem intuitive to find those beta values that would reduce the overall error sum. However, the GLM method does not effectively minimize the sum of error values (Eq. 4) since errors may contain both positive and negative values. Rather, it finds those beta values that minimize the sum of squared error values.

$$e^T e = (y - X\beta)^T (y - X\beta) \rightarrow min \tag{4}$$

Table 3: Description of Brain Regions corresponds to Channels and Source-Detector pair

			Left Channels			Right Channels
1	CH 1	S1-D1	8 - Includes Frontal eye fields	CH 55	S15-D13	8 - Includes Frontal eye f[illegible]
2	CH 2	S1-D3	9 - Dorsolateral prefrontal cortex	CH 54	S15-D11	9 - Dorsolateral prefronta[illegible]
3	CH 9	S3-D3	9 - Dorsolateral prefrontal cortex	CH 46	S13-D11	9 - Dorsolateral prefronta[illegible]
4	CH 17	S5-D3	45 - pars triangularis Broca's area	CH 37	S11-D9	45 - pars triangularis Bro[illegible]
5	CH 18	S5-D5	45 - pars triangularis Broca's area	CH 38	S11-D11	45 - pars triangularis Bro[illegible]
6	CH 4	S2-D1	6 - Pre-Motor and Supplementary Motor Cortex	CH 45	S13-D9	6 - Pre-Motor and Suppl[illegible]
7	CH 8	S3-D1	6 - Pre-Motor and Supplementary Motor Cortex	CH 47	S13-D12	6 - Pre-Motor and Suppl[illegible]
8	CH 10	S3-D4	6 - Pre-Motor and Supplementary Motor Cortex	CH 48	S13-D13	6 - Pre-Motor and Suppl[illegible]
9	CH 11	S3-D5	6 - Pre-Motor and Supplementary Motor Cortex	CH 58	S16-D13	6 - Pre-Motor and Suppl[illegible]
10	CH 5	S2-D2	4 - Primary Motor Cortex	CH 57	S16-D12	4 - Primary Motor Corte[illegible]
11	CH 6	S2-D4	4 - Primary Motor Cortex	CH 59	S16-D14	4 - Primary Motor Corte[illegible]
12	CH 13	S4-D2	40 - Supramarginal gyrus part of Wernicke's area	CH 50	S14-D10	40 - Supramarginal gyrus[illegible]
13	CH 14	S4-D4	40 - Supramarginal gyrus part of Wernicke's area	CH 51	S14-D12	40 - Supramarginal gyrus[illegible]
14	CH 15	S4-D6	40 - Supramarginal gyrus part of Wernicke's area	CH 52	S14-D14	40 - Supramarginal gyrus[illegible]
15	CH 20	S6-D4	Area between Brodmann 42L and 02L Ant. & posterior transverse temporal and Primary Somatosensory Cortex (Secondary auditory cortex)	CH 43	S12-D12	Area between 41R and b[illegible] Ant. & posterior transve[illegible] primary somatosensory c[illegible]
16	CH 21	S6-D5	43 - Subcentral area	CH 41	S12-D9	43 - Subcentral area
17	CH 25	S7-D5	48 - Retrosubicular area	CH 32	S9-D9	48 - Retrosubicular area
18	CH 22	S6-D6	22 - Superior Temporal Gyrus	CH 40	S12-D8	21 - Middle Temporal gy[illegible] 22 - Superior Temporal [illegible]
19	CH 23	S6-D7	21 - Middle Temporal gyrus, 22 - Superior Temporal Gyrus	CH 31	S9-D8	21 - Middle Temporal gy[illegible]
20	CH 26	S7-D7	21 - Middle Temporal gyrus	CH 34	S10-D8	21 - Middle Temporal gy[illegible]
21	CH 28	S8-D6	21 - Middle Temporal gyrus	CH 35	S10-D10	21 - Middle Temporal gy[illegible]
22	CH 29	S8-D7	21 - Middle Temporal gyrus	CH 42	S12-D10	22 - Superior Temporal [illegible]

The following Eq. 5 gives the optimal beta values (least squares estimates).

$$\beta = (X^T X)^{-1} X^T Y \tag{5}$$

A t-test (Eq. 6) is used to test the regression coefficient β and residual error e. The t-values are calculated by using the following formula:

$$t = \frac{c^T \beta}{\sqrt{e^2 c^T (X^T X)^{-1} c)}} \tag{6}$$

In fig. 15 show how the GLM fit of predicted response to the ΔHbO data.

References

[1] M. Izzetoglu, K. Izzetoglu, S. Bunce, H. Ayaz, A. Devaraj, B. Onaral, and K. Pourrezaei, "Functional near-infrared neuroimaging," *IEEE transactions on neural systems and rehabilitation engineering*, vol. 13, no. 2, pp. 153–159, 2005.

Table 4: The average speed of completing one Infinity walk

	C1	C2	C3	C4	C5	Average Time (sec)	Average Speed (ft/sec)
**P1**	22	20	22	22	22	21.6	1.75
**P2**	18	16	18	17	18	17.4	2.17
**P3**	18	19	18	19	17	18.2	2.07
**P4**	19	18	17	19	20	18.6	2.03
**P5**	17	18	17	17	18	17.4	2.17
**P6**	21	19	20	20	20	20	1.88

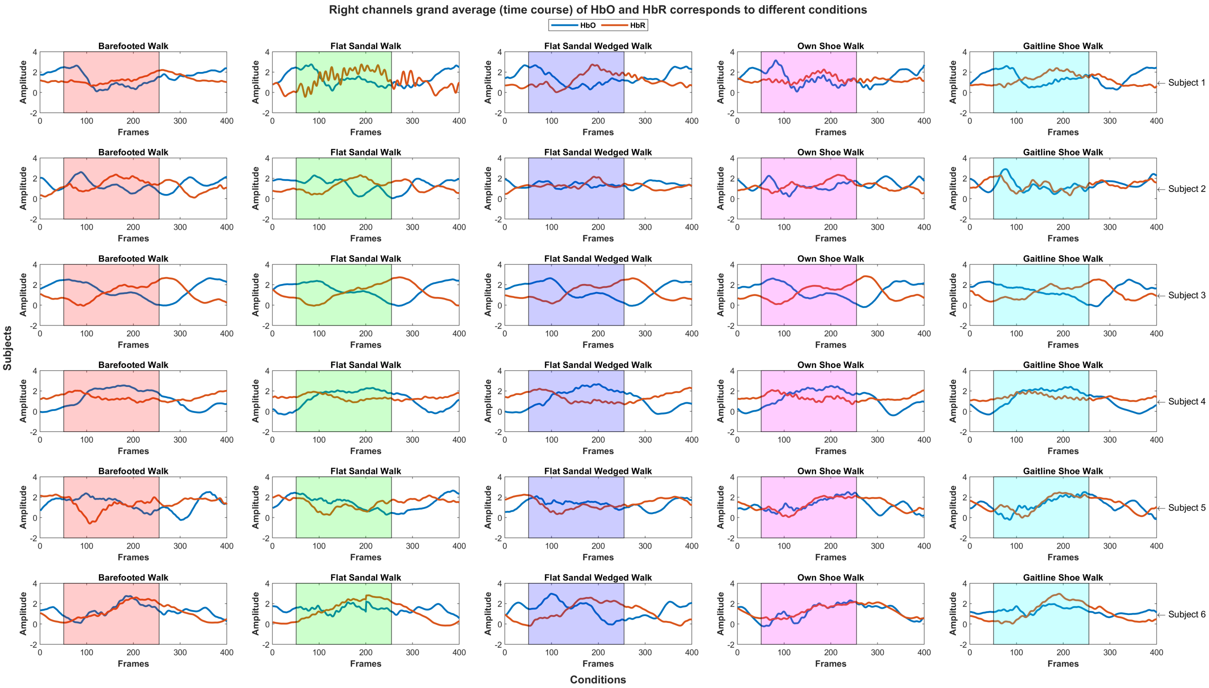

Figure 10: Grand averaging of right side channels

[2] H. Khan, N. Naseer, A. Yazidi, P. K. Eide, H. W. Hassan, and P. Mirtaheri, "Analysis of human gait using hybrid eeg-fnirs-based bci system: A review," *Frontiers in Human Neuroscience*, p. 605, 2021.

[3] X.-y. Wang, C.-c. Bao, R. An, T. Wu, D. Wang, Y.-j. Zhang, and C.-q. He, "Evaluation of the effect of physical therapy on pain and dysfunction of knee osteoarthritis based on fnirs: a randomized controlled trial protocol," *BMC Musculoskeletal Disorders*, vol. 24, no. 1, p. 152, 2023.

[4] M. Mihara and I. Miyai, "Review of functional near-infrared spectroscopy in neurorehabilitation," *Neurophotonics*, vol. 3, no. 3, pp. 031414–031414, 2016.

[5] O.-Y. Lo, M. A. Halko, J. Zhou, R. Harrison, L. A. Lipsitz, and B. Manor, "Gait speed and gait variability are associated with different functional brain networks," *Frontiers in aging neuroscience*, vol. 9, p. 390, 2017.

[6] D. Hamacher, F. Herold, P. Wiegel, D. Hamacher, and L. Schega, "Brain activity during walking: a systematic review," *Neuroscience & Biobehavioral Reviews*, vol. 57, pp. 310–327, 2015.

[7] D. Sunbeck, *Infinity walk: Preparing your mind to learn.* Jalmar Press, 1996.

[8] T. J. Szymanski, "Infinity walk: Preparing your mind to learn!," *Research and Teaching in Developmental Education*, vol. 13, no. 2, pp. 113–115, 1997.

[9] D. T. Sunbeck, ""figure-eight" track, apparatus and method for sensory-motor exercise," Oct. 3 2006. US Patent 7,115,071.

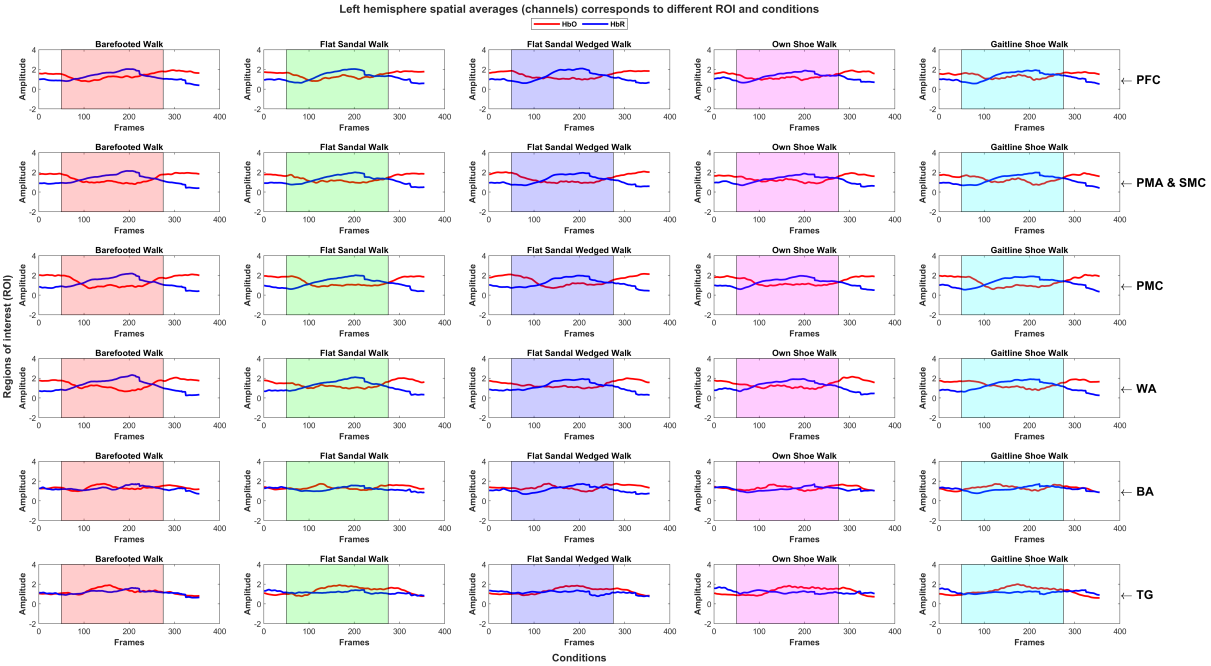

Figure 11: Grand averaging ROI on Left hemispheres: columns represent different conditions while rows represent the ROI. The shaded regions The shaded region shows the stimuli duration. Changes in amplitude are in μM. PFC: Prefrontal cortex, PMA & SMC: Pre-motor and supplementary motor cortex, PMC: Primary motor cortex, WA: Wernicke's area, BA: Broca's area, TG: Temporal gyrus. Further details about the ROI can be found in table 3

[10] D. T. Sunbeck, *Infinity Walk, Book I: The Physical Self.* Leonardo Foundation Press, 2002.

[11] T. Lyu, K. Yan, J. Lyu, X. Zhao, R. Wang, C. Zhang, M. Liu, C. Xiong, C. Liu, and Y. Wei, "Comparative efficacy of gait training for balance outcomes in patients with stroke: A systematic review and network meta-analysis," *Frontiers in Neurology*, vol. 14, 2023.

[12] V. Belluscio, E. Bergamini, M. Tramontano, R. Formisano, M. G. Buzzi, and G. Vannozzi, "Does curved walking sharpen the assessment of gait disorders? an instrumented approach based on wearable inertial sensors," *Sensors*, vol. 20, no. 18, p. 5244, 2020.

[13] R. J. Hess, J. S. Brach, S. R. Piva, and J. M. VanSwearingen, "Walking skill can be assessed in older adults: validity of the figure-of-8 walk test," *Physical therapy*, vol. 90, no. 1, pp. 89–99, 2010.

[14] C. A. Odonkor, J. C. Thomas, N. Holt, N. Latham, J. VanSwearingen, J. S. Brach, S. G. Leveille, A. Jette, and J. Bean, "A comparison of straight-and curved-path walking tests among mobility-limited older adults," *Journals of Gerontology Series A: Biomedical Sciences and Medical Sciences*, vol. 68, no. 12, pp. 1532–1539, 2013.

[15] K. Lowry, T. Woods, A. Malone, A. Krajek, A. Smiley, and J. Van Swearingen, "The figure-of-8 walk test used to detect the loss of motor skill in walking among persons with parkinson's disease," *Physiotherapy Theory and Practice*, vol. 38, no. 4, pp. 552–560, 2022.

[16] J. Schack, P. Mirtaheri, H. Steen, and T. Gjøvaag, "Assessing mobility for persons with lower limb amputation: the figure-of-eight walk test with the inclusion of two novel conditions," *Disability and Rehabilitation*, vol. 43, no. 9, pp. 1323–1332, 2021.

[17] T. Nualyong and A. Siriphorn, "Accuracy of the figure of 8 walk test with and without dual-task to predict falls in older adults," *Journal of Bodywork and Movement Therapies*, vol. 30, pp. 69–75, 2022.

[18] A.-K. Rogge, D. Hamacher, G. Cappagli, L. Kuhne, K. Hötting, A. Zech, M. Gori, and B. Röder, "Balance, gait, and navigation performance are related to physical exercise in blind and visually impaired children and adolescents," *Experimental brain research*, vol. 239, pp. 1111–1123, 2021.

[19] H. Khan, M. A. Pinto-Orellana, and P. Mirtaheri, "Brain connectivity analysis in distinct footwear conditions during infinity walk using fnirs," *Sensors*, vol. 23, no. 9, p. 4422, 2023.

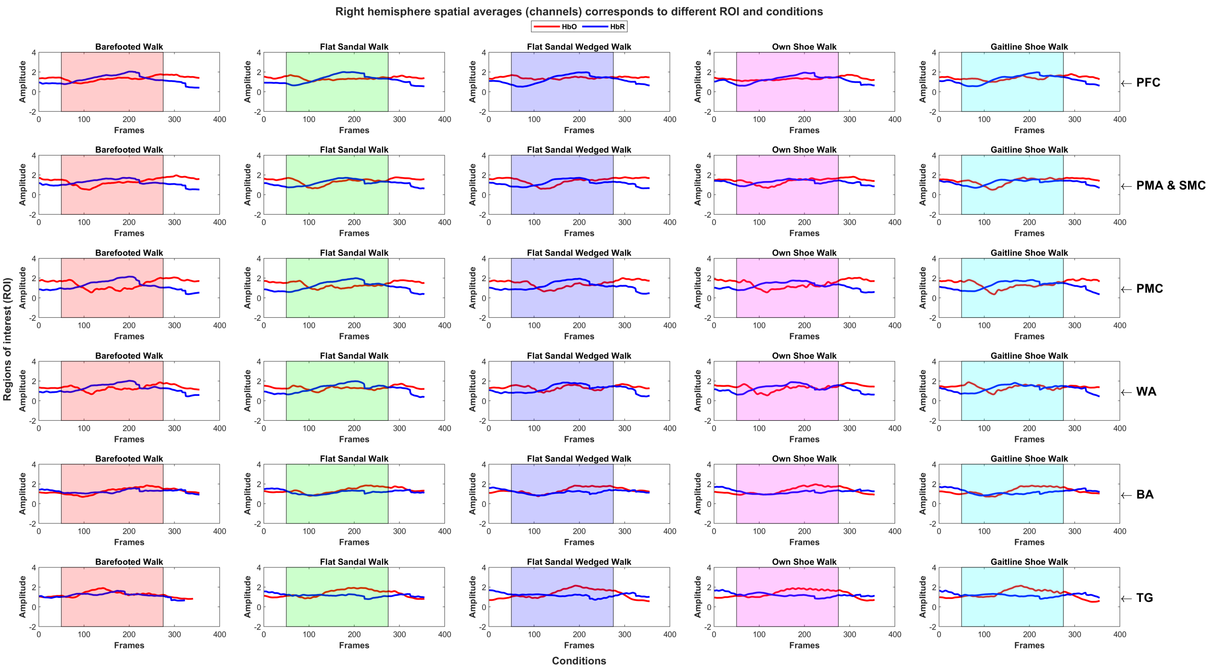

Figure 12: Grand averaging ROI on Right hemispheres: columns represent different conditions while rows represent the ROI. The shaded regions The shaded region shows the stimuli duration. Changes in amplitude are in μM. PFC: Prefrontal cortex, PMA & SMC: Pre-motor and supplementary motor cortex, PMC: Primary motor cortex, WA: Wernicke's area, BA: Broca's area, TG: Temporal gyrus. Further details about the ROI can be found in table 3

[20] Á. S. Machado, G. D. Bombach, J. Duysens, and F. P. Carpes, "Differences in foot sensitivity and plantar pressure between young adults and elderly," *Archives of gerontology and geriatrics*, vol. 63, pp. 67–71, 2016.

[21] F. B. Horak, "Postural orientation and equilibrium: what do we need to know about neural control of balance to prevent falls?," *Age and ageing*, vol. 35, no. suppl_2, pp. ii7–ii11, 2006.

[22] T. Gjovaag, P. Mirtaheri, and I. M. Starholm, "Carbohydrate and fat oxidation in persons with lower limb amputation during walking with different speeds," *Prosthetics and orthotics international*, vol. 42, no. 3, pp. 304–310, 2018.

[23] I. M. Starholm, P. Mirtaheri, N. Kapetanovic, T. Versto, G. Skyttemyr, F. T. Westby, and T. Gjovaag, "Energy expenditure of transfemoral amputees during floor and treadmill walking with different speeds," *Prosthetics and orthotics international*, vol. 40, no. 3, pp. 336–342, 2016.

[24] N. Seethapathi and M. Srinivasan, "The metabolic cost of changing walking speeds is significant, implies lower optimal speeds for shorter distances, and increases daily energy estimates," *Biology letters*, vol. 11, no. 9, p. 20150486, 2015.

[25] G. A. Z. Morais, J. B. Balardin, and J. R. Sato, "fnirs optodes' location decider (fold): a toolbox for probe arrangement guided by brain regions-of-interest," *Scientific reports*, vol. 8, no. 1, pp. 1–11, 2018.

[26] V. Fonov, A. C. Evans, K. Botteron, C. R. Almli, R. C. McKinstry, and D. L. Collins, "Unbiased average age-appropriate atlases for pediatric studies," *NeuroImage*, vol. 54, no. 1, pp. 313–327, 2011.

[27] L. Pollonini, C. Olds, H. Abaya, H. Bortfeld, M. S. Beauchamp, and J. S. Oghalai, "Auditory cortex activation to natural speech and simulated cochlear implant speech measured with functional near-infrared spectroscopy," *Hearing research*, vol. 309, pp. 84–93, 2014.

[28] G. A. Z. Morais, F. Scholkmann, J. B. Balardin, R. A. Furucho, R. C. V. de Paula, C. E. Biazoli, and J. R. Sato, "Non-neuronal evoked and spontaneous hemodynamic changes in the anterior temporal region of the human head may lead to misinterpretations of functional near-infrared spectroscopy signals," *Neurophotonics*, vol. 5, no. 1, p. 011002, 2017.

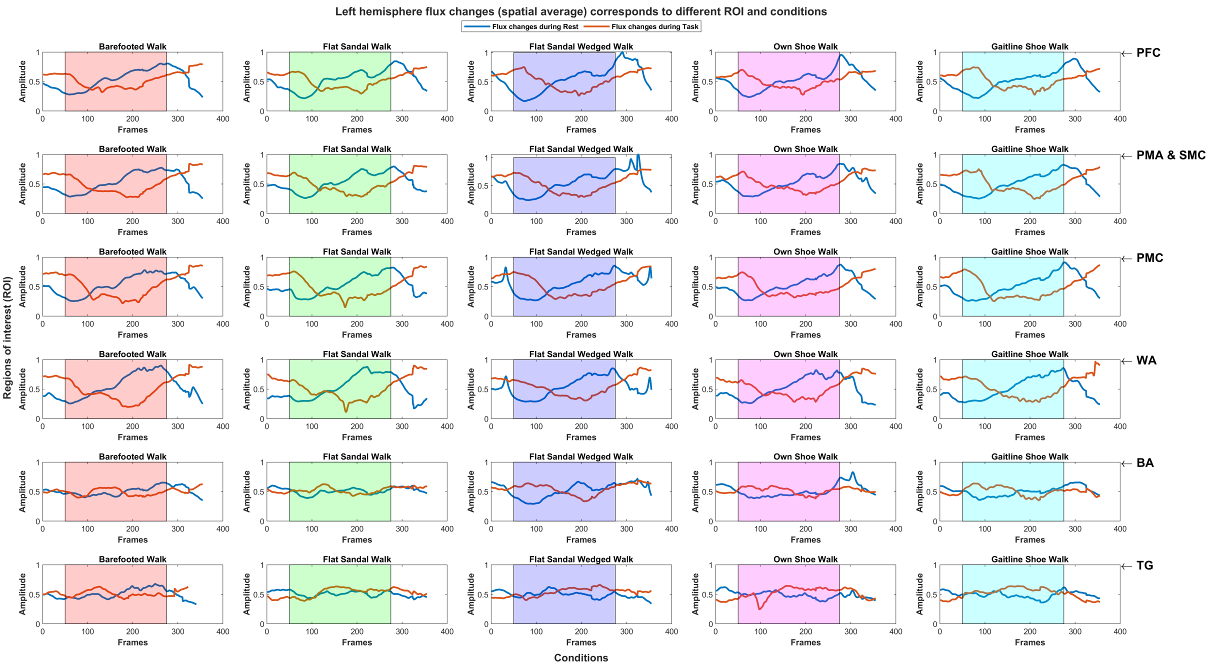

Figure 13: Blood flow changes over ROI in left hemispheres: columns represent different conditions while rows represent the ROI. The shaded regions The shaded region shows the stimuli duration. Changes in amplitude are in μM. PFC: Prefrontal cortex, PMA & SMC: Pre-motor and supplementary motor cortex, PMC: Primary motor cortex, WA: Wernicke's area, BA: Broca's area, TG: Temporal gyrus. Further details about the ROI can be found in table 3

[29] G. A. Zimeo Morais, F. Scholkmann, J. B. Balardin, R. A. Furucho, R. C. V. de Paula, C. E. Biazoli Jr, and J. R. Sato, "Non-neuronal evoked and spontaneous hemodynamic changes in the anterior temporal region of the human head may lead to misinterpretations of functional near-infrared spectroscopy signals," *Neurophotonics*, vol. 5, no. 1, pp. 011002–011002, 2018.

[30] J. van Brakel, "Robust peak detection algorithm (using z-scores)," *Stack Overflow: New York, NY, USA*, 2014.

[31] M. Steffen, "A simple method for monotonic interpolation in one dimension," *Astronomy and Astrophysics, Vol. 239, NO. NOV (II), P. 443, 1990*, vol. 239, p. 443, 1990.

[32] F. A. Fishburn, R. S. Ludlum, C. J. Vaidya, and A. V. Medvedev, "Temporal derivative distribution repair (tddr): a motion correction method for fnirs," *Neuroimage*, vol. 184, pp. 171–179, 2019.

[33] H. Yamaguchi, H. Yamauchi, S. Hazama, H. Hamamoto, and N. Inoue, "Correlation between cerebral oxygen metabolism and cerebral blood flow simultaneously measured before and after acetazolamide administration," *Journal of Biomedical Optics*, vol. 4, no. 4, pp. 418–423, 1999.

[34] M. McNarry, R. Wilson, M. Holton, I. Griffiths, and K. Mackintosh, "Investigating the relationship between energy expenditure, walking speed and angle of turning in humans," *PLoS One*, vol. 12, no. 8, p. e0182333, 2017.

[35] H. Khan, H. Nazeer, H. Engell, N. Naseer, O. Korostynska, and P. Mirtaheri, "Prefrontal cortex activation measured during different footwear and ground conditions using fnirs—a case study," in *2021 International Conference on Artificial Intelligence and Mechatronics Systems (AIMS)*, pp. 1–6, IEEE, 2021.

[36] H. Khan, N. K. Qureshi, A. Yazidi, H. Engell, and P. Mirtaheri, "Single-leg stance on a challenging surface can enhance cortical activation in the right hemisphere-a case study," *Heliyon*, 2023.

[37] L. Saxby, "Proprioception, making sense of barefoot running," *Terra Plana International*, pp. 1–32, 2011.

[38] A. von Lühmann, A. Ortega-Martinez, D. A. Boas, and M. A. Yücel, "Using the general linear model to improve performance in fnirs single trial analysis and classification: a perspective," *Frontiers in human neuroscience*, vol. 14, 2020.

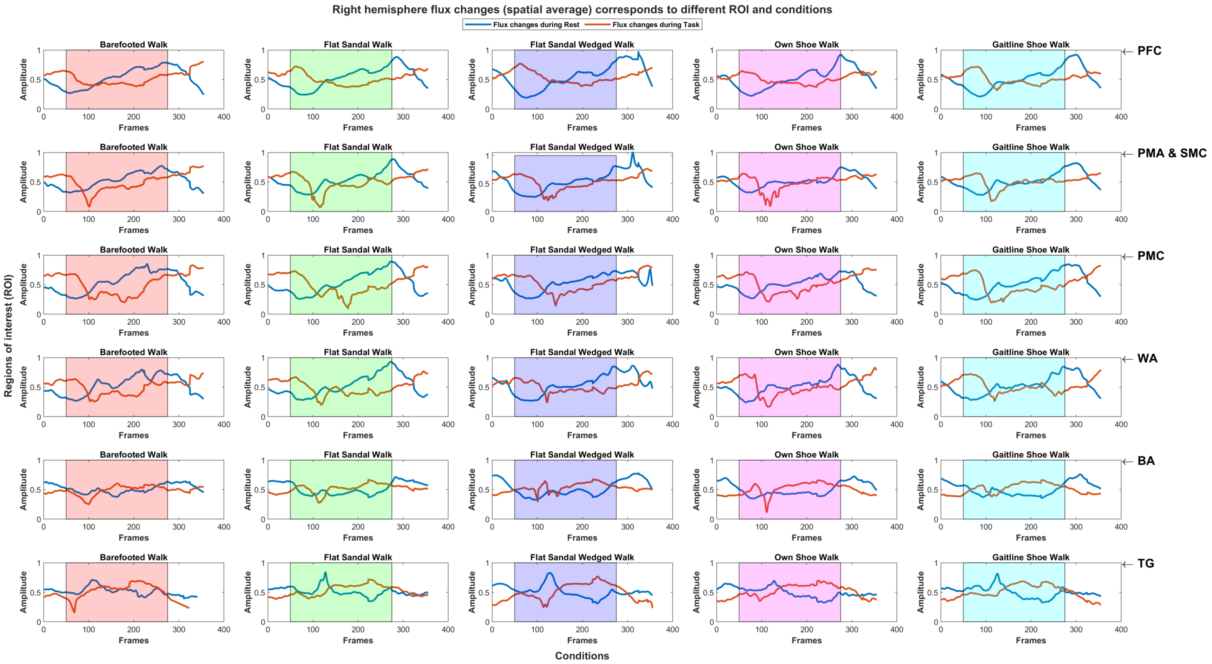

Figure 14: Blood flow changes over ROI in right hemispheres: columns represent different conditions while rows represent the ROI. The shaded regions The shaded region shows the stimuli duration. Changes in amplitude are in μM. PFC: Prefrontal cortex, PMA & SMC: Pre-motor and supplementary motor cortex, PMC: Primary motor cortex, WA: Wernicke's area, BA: Broca's area, TG: Temporal gyrus. Further details about the ROI can be found in table 3

[39] M. Uga, I. Dan, T. Sano, H. Dan, and E. Watanabe, "Optimizing the general linear model for functional near-infrared spectroscopy: an adaptive hemodynamic response function approach," *Neurophotonics*, vol. 1, no. 1, p. 015004, 2014.

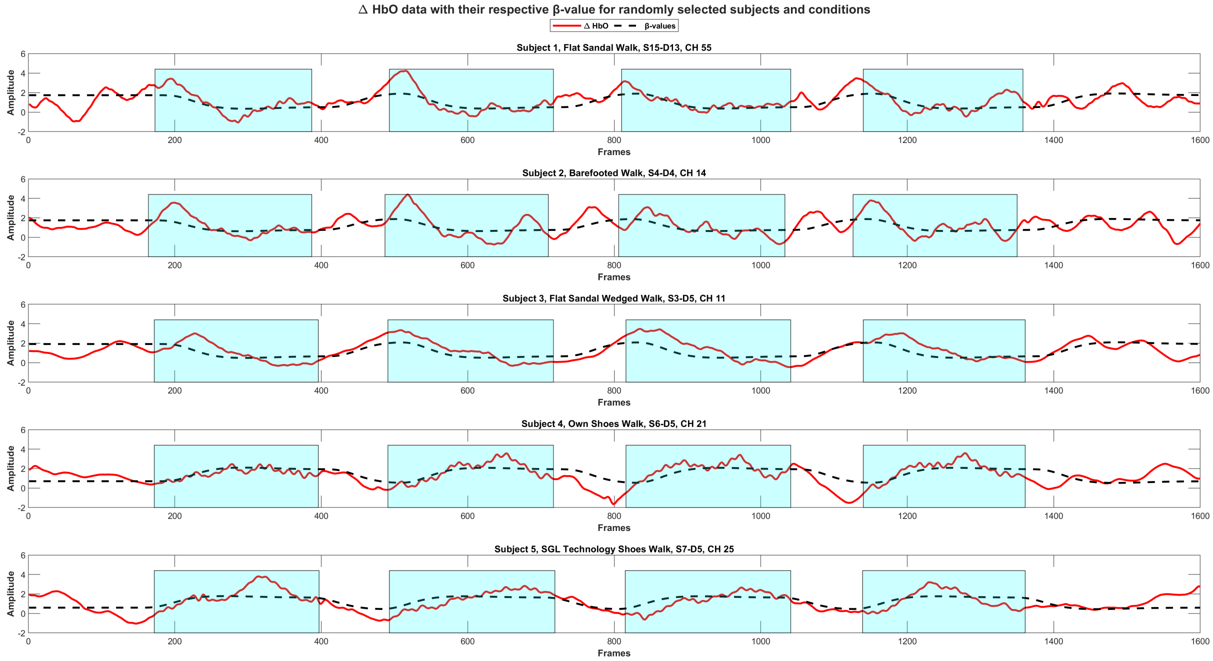

Figure 15: GLM fitted curve (β-values) to Δ HbO data

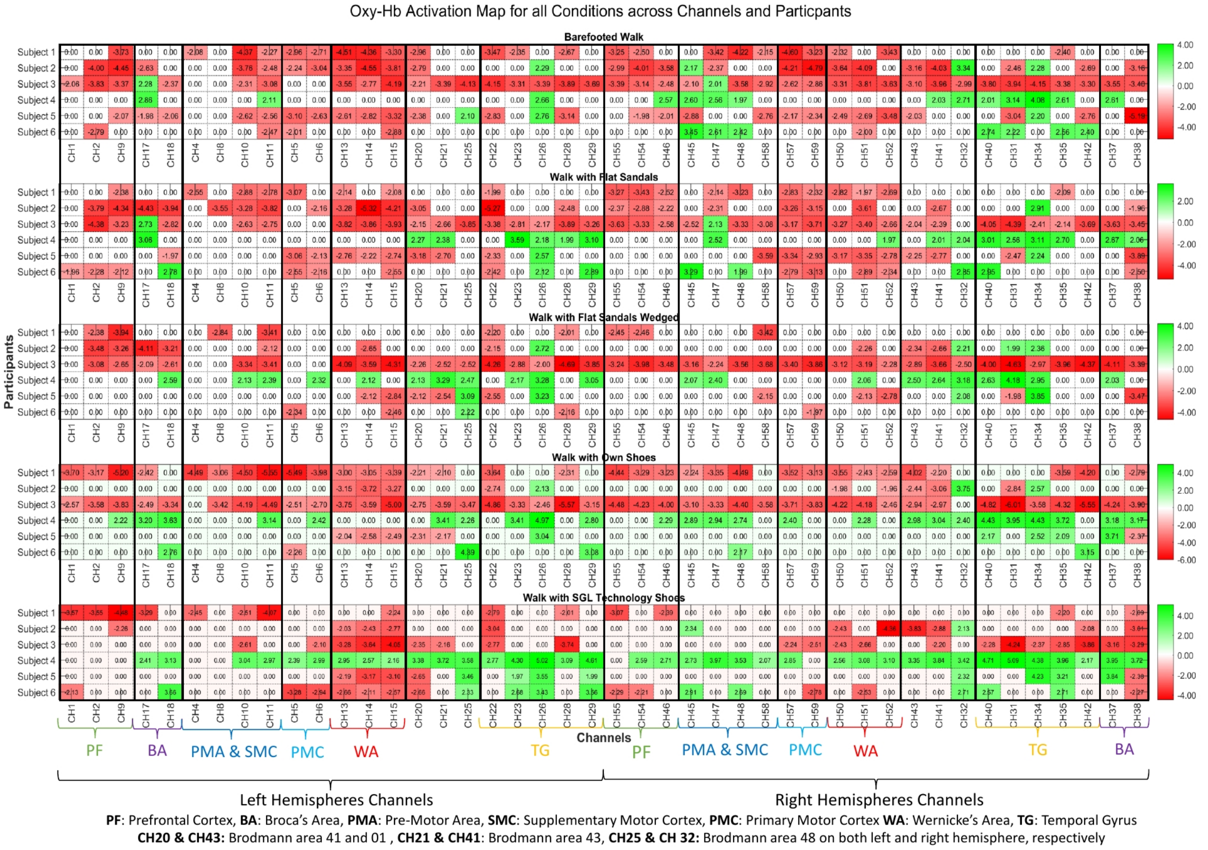

Figure 16: Δ HbO activation map for significantly (p-value < 0.05) active channels. For the sake of simplicity, non-significant ($p \geq 0.05$) channels value are shown as zero in the heat map.

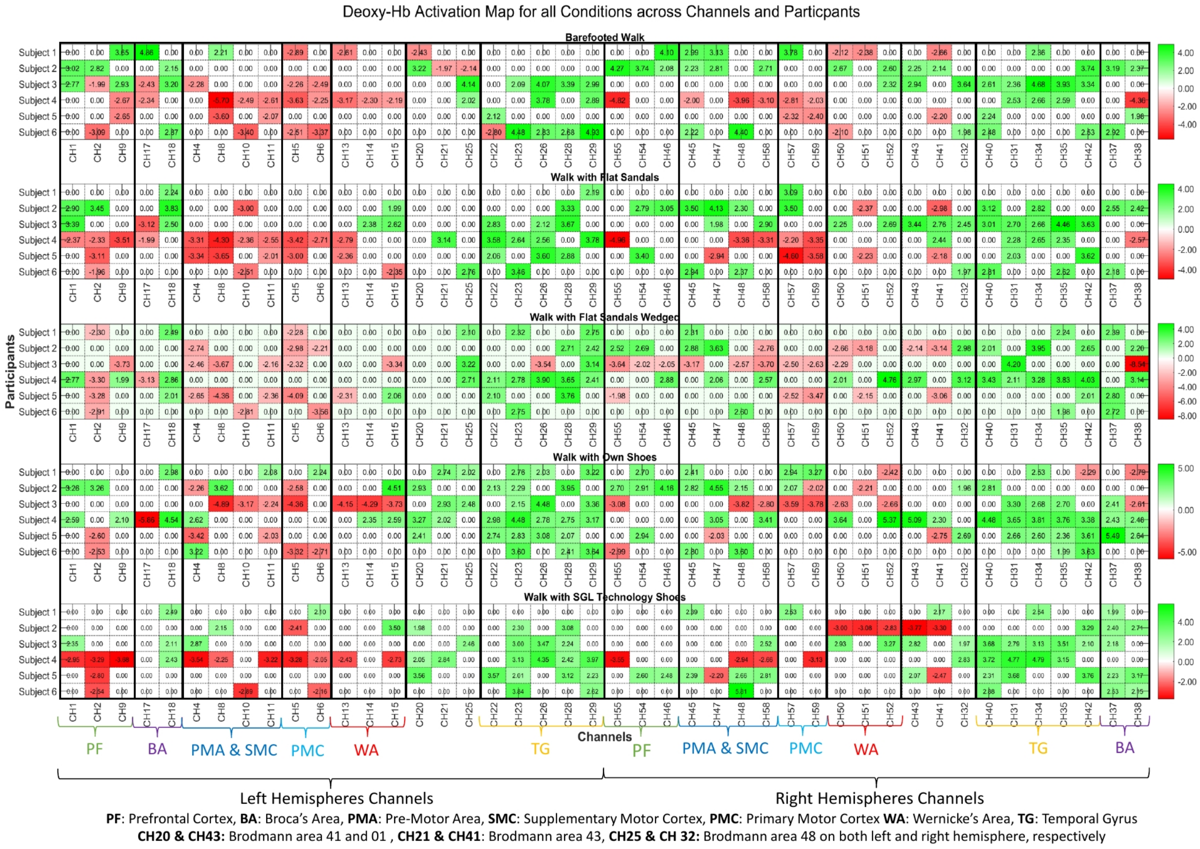

Figure 17: Δ HbR activation map for significantly (p-value $<$ 0.05) active channels
